# Snakebite knowledge among healthcare workers in Gabon: A health facility-based cross-sectional survey

**DOI:** 10.1371/journal.pntd.0013742

**Published:** 2026-03-16

**Authors:** Jade D. Rae, Rica Artus, Friederike Hunstig, Ghyslain Mombo-Ngoma, Alex Hounmenou Zinsou, Dearie Glory Okwu, Wilfrid Ndzebe Ndoumba, Rella Zoleko Manego, Michael Ramharter, Bertrand Lell, Peter Gottfried Kremsner, Jörg Blessmann, Benno Kreuels

**Affiliations:** 1 Research Group Neglected Diseases and Envenoming, Bernhard Nocht Institute for Tropical Medicine, Hamburg, Germany; 2 Centre de Recherches Médicales de Lambaréné, Lambaréné, Gabon; 3 Division of Infectiology, Outpatient Center, University Medical Center Hamburg-Eppendorf, Hamburg, Germany; 4 Research Group Drug Implementation, Bernhard Nocht Institute for Tropical Medicine, I. Department of Medicine, University Medical Center Hamburg-Eppendorf, Hamburg, Germany; 5 German Center for Infection Research (DZIF), Partner Site Hamburg-Lübeck-Borstel-Riems, Hamburg, Germany; 6 Center for Tropical Medicine, Bernhard Nocht Institute for Tropical Medicine and I. Department of Medicine, University Medical Center Hamburg-Eppendorf, Hamburg, Germany; 7 Institute of Tropical Medicine, University of Tübingen, Tübingen, Baden-Württemberg, Germany; 8 Division of Infectious Diseases and Tropical Medicine, I. Department of Medicine, Medical University of Vienna, Vienna, Austria; 9 German Center for Infection Research (DZIF), Partner Site Tübingen, Tübingen, Baden-Württemberg, Germany; 10 Division for Tropical Medicine, I. Department of Medicine, University Medical Center Hamburg-Eppendorf, Hamburg, Germany; Fundação de Medicina Tropical Doutor Heitor Vieira Dourado: Fundacao de Medicina Tropical Doutor Heitor Vieira Dourado, BRAZIL

## Abstract

**Background:**

Snakebite envenoming remains a neglected health issue in many countries, including Gabon, where the limited availability of snakebite-specific training, clinical guidelines, and essential resources at health facilities may lead to gaps in healthcare workers’ knowledge and confidence and the use of non-recommended treatment practices. This study aimed to assess healthcare workers’ knowledge of snakebite management in the Ogooué et des Lacs department of Moyen-Ogooué province in Gabon.

**Methods:**

From June to August 2023, we conducted a cross-sectional survey targeting all healthcare workers in Ogooué et des Lacs who may be involved in snakebite management. We collected information on prior training, self-perceived knowledge of snakebite management, symptom recognition, clinical management practices, and snake identification. Knowledge was assessed using 10 true/false symptom questions and 12 management questions, with one point assigned for each correct response. Generalised linear binomial regression modelling was used to evaluate associations between knowledge and participant characteristics.

**Results:**

A total of 171 healthcare workers (78% of those eligible) participated. Overall, 40% (68/171) scored below 50% on symptom questions, and 66% (113/171) scored below 50% on management questions. Inappropriate practices were frequently recommended, including tourniquet use (70%, 120/171) and venom aspiration (73%, 125/171). Nurse assistants and nurses had a lower odds of correct responses than doctors (OR: 0.68, 95% confidence interval (CI): 0.55-0.84, and OR: 0.78, 95% CI: 0.62-0.98, respectively), while healthcare workers at secondary health facilities performed better than those at primary health facilities (OR: 1.23, 95% CI: 1.04-1.46). Venomous snakes were correctly identified as such in 74% (109/148) of responses, compared with 28% (42/148) for non-venomous snakes.

**Conclusion:**

Gaps in snakebite knowledge and management were identified among healthcare workers in the study region. Targeted training, national clinical guidelines, and improved access to antivenom are needed to strengthen snakebite management in Ogooué et des Lacs and potentially more broadly in Gabon.

## Author summary

Snakebite envenomation remains a neglected public health issue in many countries, including Gabon, where healthcare workers often face significant gaps in the training and resources needed for effective case management. In this study, we aimed to survey all healthcare workers in the department of Ogooué et des Lacs in the Moyen-Ogooué province of Gabon who, in their current role, could be involved in snakebite management. We assessed prior snakebite training and experience and evaluated knowledge of snakebite symptoms, treatment practices, local snake species identification, and familiarity with existing clinical guidelines. Using a structured questionnaire, we found that 40% of respondents scored below 50% on questions related to snakebite symptoms, and two-thirds scored below 50% on management practices. Many health workers also struggled to identify venomous snakes from photographs. These findings highlight important gaps in snakebite knowledge among healthcare workers in this setting and underscore the need for comprehensive and ongoing training programs and the development of standardised national guidelines for snakebite management. Strengthening healthcare workers’ knowledge and clinical competencies in this area could improve the clinical outcomes for snakebite patients in Gabon.

## Introduction

Snakebite envenoming is a significant yet often under-recognised public health issue, particularly in tropical and subtropical low- and middle-income countries (LMICs) where human-snake interactions are common. Despite being added to the World Health Organisation (WHO) list of neglected tropical diseases (NTDs) in 2017, and the WHO Snakebite Envenoming Strategy announced in 2019 to reduce deaths and disabilities by 50% by 2030 [1], the true burden of snakebite envenoming remains poorly understood in many countries due to a lack of epidemiological data. Regional estimates, however, offer insights into the scale of the problem. For example, in sub-Saharan Africa, an estimated 314,000 snakebite cases are seen at health facilities each year, resulting in more than 7,000 reported deaths [2]. These figures likely underestimate the true burden, as many victims do not seek formal healthcare and instead rely on traditional healers [3–6].

Traditional treatments provided outside the formal healthcare system, though lacking evidence of efficacy [7], are often preferred and trusted by snakebite victims over modern medicine, particularly in rural areas of Africa and Asia [8–12]. This preference, in part, likely stems from systematic weaknesses in formal healthcare systems in these regions, such as the limited availability of antivenom, insufficient training of healthcare workers in snakebite management, and inappropriate or delayed treatment practices [6,13–15]. Addressing these challenges requires not only improved access to essential resources, such as effective antivenom, but also strengthening the capacity and knowledge of healthcare workers [16,17].

In Gabon, several medically important venomous snake species can be found [18,19]. The most likely relevant snake species include the Gaboon viper (*Bitis gabonica)*, which causes mainly cytotoxic envenoming, with case reports describing severe hemotoxic envenoming [20], and the forest cobra (*Naja melanoleuca)* and Jameson’s mamba (*Dendroaspis jamesoni*), which both cause neurotoxic envenoming [21]. However, very little is known about the number of envenomations caused by venomous snakes in the country. Until recently, no data on the epidemiology and clinical management of snakebites in Gabon had been published due to a lack of official data on snakebite cases, morbidity and mortality. To address this gap, we recently conducted household surveys in the department of Ogooué et des Lacs, Gabon [22]. The estimated incidence of snakebites was 246 cases per 100,000 person-years, with approximately 18% of cases reporting signs of envenoming, and 60% of snakebite patients seeking care at a health facility [22]. Although 81% of patients who went to a health facility received antivenom, it was frequently underdosed, and the use of unrecommended treatments was common, including the administration of steroids (51%) and antibiotics (41%) [22]. These findings highlight the deficiencies in the clinical management of snakebites and point to potential gaps in health workers’ knowledge and skills in evidence-based treatment protocols.

In light of these findings, the present study assesses snakebite knowledge, self-perceived snakebite management knowledge, and recognition of local snake species through a cross-sectional survey designed to include all healthcare workers who, in their current role, may be involved in snakebite case management in the department of Ogooué et des Lacs, Gabon. By identifying key misconceptions and knowledge gaps, the results of this study have the potential to inform strategies to strengthen clinical practice, improve decision-making, and support the development of context-appropriate tools for snakebite management in Gabon and similar settings.

## Methods

### Ethics statement

Written informed consent was obtained from every study participant. The study was evaluated and approved by the Institutional Ethics Committee of the Centre de Recherches Médicales de Lambaréné (CERMEL) (CEI-010/2022) in Gabon and by the Medical Association in Hamburg (Ärztekammer Hamburg) in Germany (2023–300326-WF).

### Study design and setting

Between June and August 2023, we conducted a cross-sectional survey to describe and evaluate snakebite knowledge among all healthcare workers who, in their current role, could be involved in snakebite case management at health facilities in the centrally located department of Ogooué et des Lacs, in the Moyen-Ogooué province of Gabon. This study area was chosen for its proximity to ongoing research activities led by Centre de Recherches Médicales de Lambaréné (CERMEL) and because it is the only area in Gabon where previous studies on snakebites have been conducted. This manuscript was prepared in accordance with the STROBE (Strengthening the Reporting of Observational Studies in Epidemiology) statement for cross-sectional studies (S1 Data).

### Study population

There are 29 health facilities in Ogooué et des Lacs that provide health services, including two secondary health facilities (Hôpital Albert Schweitzer and Centre Hospitalier Régional Georges Rawiri de Lambaréné) and 27 primary health facilities (22 dispensaries, four medical centres, and one outpatient clinic). We aimed to survey all healthcare workers at these health facilities who, in their current role, may be involved in the clinical management of snakebites. This included doctors, nurses, and nurse assistants (who always work alongside a nurse or doctor) working in the hospitals’ medicine, paediatrics, surgery, and emergency departments, as well as at dispensaries, medical centres, and the outpatient clinic identified from facility staffing lists. Anyone not involved in the clinical management of snakebite cases, or still in training (medical or nursing students), was excluded from the survey.

### Data collection

We adapted a questionnaire on snakebite knowledge and practices previously used in Laos [23] to reflect the snake species and clinical practices specific to the study area through consultation with local staff [21]. The questionnaire was then translated into French by one of the study team members (RA). This translation was then checked independently by a proficient French speaker (FH) and a native speaker (AHZ). The resulting questionnaire was piloted in a group of four health workers in the department (two nurses, one nurse assistant, and one doctor). Based on feedback received during the pilot testing, more details were included in some questions (e.g., what is meant by neurotoxic). The adapted questionnaire is provided in S1 File.

The questionnaire was divided into four sections (A - D). Section A collected information on healthcare worker demographics, prior training and experience in managing snakebite cases, awareness of clinical guidelines and sources of clinical support, and self-perceived knowledge of snakebite management. Sections B and C each contained 12 true/false questions designed to assess knowledge of snakebite symptoms (Section B) and management practices (Section C). In Section D, healthcare workers were asked whether they consented to see photos of snakes for identification. Those who agreed were shown photos of 12 local snake species (found in and around the department) and asked to name each and classify them as venomous or non-venomous.

To recruit healthcare workers for the survey, we conducted three visits to the secondary health facilities on different days and shifts and made appointments to survey staff at the primary health facilities. To reduce the risk of social desirability bias, surveyed individuals completed the questionnaire independently, either on paper or on an electronic tablet. No participant names were recorded. The survey respondents were told to leave any questions they did not know the answer to blank.

Data were collected, entered, and managed using Research Electronic Data Capture (REDCap) (version 12.5.6), hosted at the Bernhard Nocht Institute for Tropical Medicine (BNITM) [24,25].

### Data analysis

Continuous variables were summarised using the median (25^th^ - 75^th^ percentiles), while categorical variables were summarised using percentages (proportions). Healthcare workers who did not complete all questions were included in the analysis. However, missing responses (except for those to knowledge questions) were excluded from the calculation of results. Healthcare worker characteristics, including sex, age, workplace (primary or secondary health facility), years of clinical experience, snakebite training, snakebites treated, and self-perceived snakebite management knowledge, were summarised by profession.

For knowledge assessments, one point was assigned for each correct answer, and zero points were assigned for incorrect or blank (“don’t know”) responses. Knowledge scores were calculated for each healthcare worker as the percentage of correct responses to questions on snakebite symptoms and management separately. Scores were split into quartiles (0–25%, 26–50%, 51–75%, and 76–100% correct responses) and summarised by profession. The correct responses to two questions on snakebite symptoms were unclear due to a lack of consistent evidence. These questions were not included in the calculation of symptom or overall knowledge scores. An overall knowledge score was calculated by combining the scores for symptoms and management. Using complete-case data, generalised linear binomial regression models were used to examine associations between healthcare worker characteristics and the odds of providing a correct response to knowledge assessment questions. Univariate analyses were conducted for each covariate, followed by a multivariate model including age, profession, health facility type (primary or secondary), snakebite management training, years of clinical work experience, experience treating snakebite cases, and self-perceived snakebite management knowledge. The inclusion of a random intercept for individual health facilities was considered, but it did not capture the variability in the outcome better than the fixed-effects-only model.

The snake names provided in Section D were classified as correct if the appropriate local, common, or scientific name was provided. The names and venomous status are provided in Table A in S1 File. For instance, a response that included “Moudouma” (local name), “Cobra” (common name), or “*Naja*” or “*Naja melanoleuca*” (scientific name) was considered correct for *Naja* *melanoleuca*. The knowledge of snake names and venomous status was summarised as the percentage of correct, incorrect, and “don’t know” responses for each snake.

Awareness of clinical guidelines and known sources of clinical support, including where to transfer snakebite patients, was summarised by health facility type.

All statistical analyses were performed in R (version 4.3.1) [26].

## Results

Between June and August 2023, we surveyed 171 healthcare workers across 28 health facilities in Ogooué et des Lacs, Gabon (one dispensary could not be visited). This represents 78% (171/220) of the estimated eligible healthcare workers in the department’s secondary (81%, 123/151) and primary (70%, 48/69) health facilities. The majority of healthcare workers were female (67%, 115/171), while males accounted for the majority of doctors (61%, 19/31) (Table 1). Most surveyed healthcare workers (72%, 123/171) were employed at a secondary health facility at the time of the survey, and 72% (113/157) had six or more years of clinical work experience (Table 1).

**Table 1:** Characteristics of the surveyed healthcare workers.

		Overall	Doctor	Nurse	Nurse assistant
		% (n/N)^1^; Median [25^th^ - 75^th^ percentiles]			
Total		100 (171/171)	18.1 (31/171)	29.8 (51/171)	52.0 (89/171)
Sex	Female	67.3 (115/171)	38.7 (12/31)	51.0 (26/51)	86.5 (77/89)
	Male	32.7 (56/171)	61.3 (19/31)	49.0 (25/51)	13.5 (12/89)
Age (years)		42 [33 - 49]	36 [32 - 44]	47 [38 - 51]	41 [32 - 49]
Age group	20 - 29	14.5 (24/165)	22.6 (7/31)	10.2 (5/49)	14.1 (12/85)
	30 - 39	28.5 (47/165)	38.7 (12/31)	16.3 (8/49)	31.8 (27/85)
	40 - 49	36.4 (60/165)	25.8 (8/31)	42.9 (21/49)	36.5 (31/85)
	≥ 50	20.6 (34/165)	12.9 (4/31)	30.6 (15/49)	17.6 (15/85)
Health facility	Primary	28.1 (48/171)	22.6 (7/31)	31.4 (16/51)	28.1 (25/89)
	Secondary	71.9 (123/171)	77.4 (24/31)	68.6 (35/51)	71.9 (64/89)
Clinical work experience (years)	≤ 1	7.6 (12/157)	0.0 (0/30)	8.2 (4/49)	10.3 (8/78)
	2 - 5	20.4 (32/157)	33.3 (10/30)	14.3 (7/49)	19.2 (15/78)
	6 - 10	21.0 (33/157)	43.3 (13/30)	12.2 (6/49)	17.9 (14/78)
	> 10	51.0 (80/157)	23.3 (7/30)	65.3 (32/49)	52.6 (41/78)

^1^ Missing responses for some individuals resulted in varying denominators.

Overall, 53% (90/171) of healthcare workers had received snakebite management training, with 44% (75/171) receiving training during their degree for a median (25^th^ - 75^th^ percentiles) of 2 hours (2– 4) and 19% (33/170) receiving post-graduation training for a median of 2 hours (1– 2). Those who received training during their degree only (without post-graduation training) accounted for 33% (56/170) of healthcare workers. By profession, a slightly higher percentage of doctors (65%, 20/31) received snakebite training compared to nurses (59%, 30/51) and nurse assistants (45%, 40/89). The most common topic of training was snakebite treatment (43%, 73/171), with approximately half of all doctors and nurses trained in this area (Table 2). A summary of the training topics covered during studies or post-graduation is presented in Table A in S2 File. Half of all healthcare workers had managed a snakebite case. Of the healthcare workers who had experience treating snakebites, those with less than one year of clinical work experience had treated a median (25^th^ - 75^th^ percentiles) of 3.5 cases (3– 4) compared with a median of 4 (2– 7) cases treated by those with more than 10 years of clinical work experience.

**Table 2:** Healthcare worker snakebite training, experience, and knowledge.

		Overall	Doctor	Nurse	Nurse assistant
		% (n/N)^1^; Median [25^th^ - 75^th^ percentiles]			
Received snakebite training^2^	During studies	43.9 (75/171)	61.3 (19/31)	52.9 (27/51)	32.6 (29/89)
	Post-graduate	19.4 (33/170)	25.8 (8/31)	10.0 (5/50)	22.5 (20/89)
Training topic^3^	Snakes, snake venom, and envenoming syndromes	26.3 (45/171)	45.2 (14/31)	25.5 (13/51)	20.2 (18/89)
	Prevention	23.4 (40/171)	29.0 (9/31)	27.5 (14/51)	19.1 (17/89)
	Diagnosis	28.7 (49/171)	45.2 (14/31)	31.4 (16/51)	21.3 (19/89)
	Treatment	42.7 (73/171)	51.6 (16/31)	49.0 (25/51)	36.0 (32/89)
	Adverse antivenom reactions	16.4 (28/171)	25.8 (8/31)	15.7 (8/51)	13.5 (12/89)
Treated a snakebite		50.9 (84/165)	64.5 (20/31)	45.8 (22/48)	48.8 (42/86)
Number of snakebites treated		3 [2 - 5]	2.5 [2 - 4.25]	4 [2.25 - 6.5]	2 [1 - 4]
Most recent snakebite case treated^4^	< 1 year ago	32.9 (26/79)	30.0 (6/20)	30.0 (6/20)	35.9 (14/39)
	1 - 5 years ago	34.2 (27/79)	40.0 (8/20)	40.0 (8/20)	28.2 (11/39)
	> 5 years ago	32.9 (26/79)	30.0 (6/20)	30.0 (6/20)	35.9 (14/39)
Knowledge score (%)^5^	Symptoms	60 [50, 70]	60 [55, 70]	60 [50, 70]	60 [50, 60]
	Management	50 [42 - 58]	58 [46 - 75]	50 [42 - 58]	42 [33 - 58]

^1^ Missing responses for some individuals resulted in varying denominators.

^2^ An individual can be counted in both the training received during studies and the training received post-graduation.

^3^ Training topics were summarised across the training received during studies and post-graduation.

^4^ The most recent snakebite case treated was summarised for those who had treated at least one snakebite.

^5^ Knowledge scores were calculated as the percentage of correct responses to 10 questions on symptoms and 12 questions on management.

**Fig 1:**
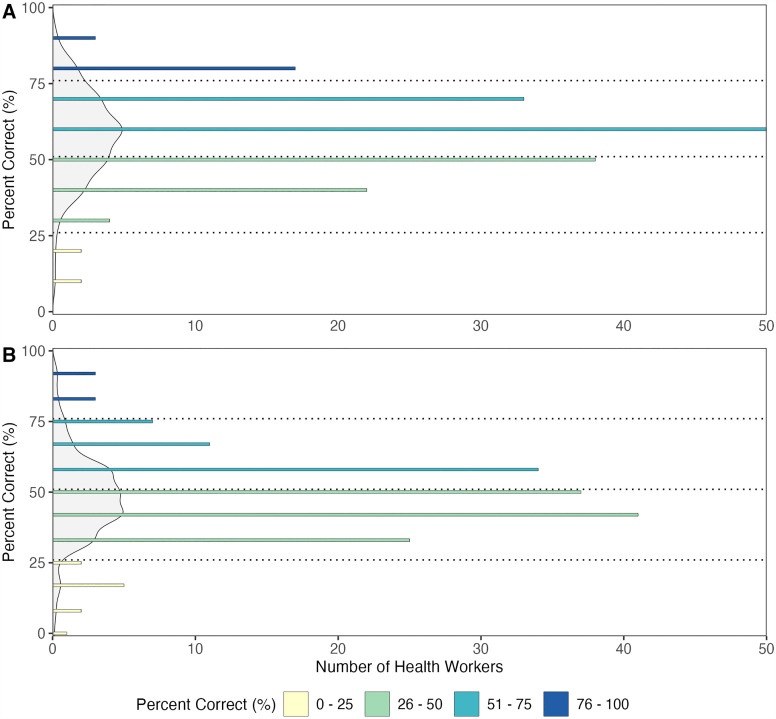
Number of healthcare workers by knowledge score on snakebite (A) symptoms and (B) management. Knowledge scores were calculated as the percentage of correct responses to 10 questions on snakebite symptoms and 12 questions on management. Each possible score is coloured according to the quartile range it falls within (pale yellow: 0 - 25%, pale green: 26 - 50%, mid blue: 51 - 75%, and dark blue: 76 - 100%). The light grey area represents the density of healthcare workers. The dotted horizontal lines represent the quartiles of knowledge scores.

In snakebite symptom knowledge assessments, 12% (20/171) of healthcare workers scored above 75%, 48% (83/171) scored between 51% and 75%, and 40% (68/171) scored below 50% (Fig 1A). By profession, a higher percentage (48%, 43/89) of nurse assistants scored below 50% compared to nurses (33%, 17/51) and doctors (26%, 8/31). In snakebite management knowledge assessments, 4% (6/171) of healthcare workers scored above 75%, 30% (52/171) scored between 51% and 75%, and 66% (113/171) scored below 50% (Fig 1B). By profession, a higher percentage (74%, 66/89) of nurse assistants scored below 50% compared to nurses (67%, 34/51) and doctors (42%, 13/31).

The majority of healthcare workers incorrectly answered questions on the specific symptoms associated with bites from snake species common in the area. Cytotoxic lesions and necrosis were incorrectly identified as common symptoms following bites from a Blanding’s tree snake (70%, 119/171) and systemic bleeding was incorrectly identified as a common symptom following bites from a forest cobra (63%, 107/171). Item-level performance on the knowledge assessment question is provided in Table B in S2 File.

For each question on snakebite management, a higher percentage of doctors answered correctly when compared with nurses and nurse assistants, aside from one question: 29% (9/31) of doctors correctly answered that children require the same antivenom dose as adults, compared with 41% (21/51) of nurses and 43% (38/89) of nurse assistants (Table B in S2 File). Irrespective of profession, the majority of healthcare workers were unaware that certain practices were inappropriate. For example, tourniquets were recommended by 70% (120/171), and venom aspiration was recommended by 73% (125/171) (Table B in S2 File).

After adjusting for other factors, nurse assistants had 32% lower odds of providing a correct response to knowledge questions compared with doctors (OR: 0.68, 95% confidence interval (CI): 0.55 - 0.84), while nurses had a 22% lower odds of providing a correct response compared with doctors (OR: 0.78, 95% CI: 0.62 - 0.98). The odds of a correct response were higher among health workers at secondary health facilities than among those at primary health facilities (OR: 1.23, 95% CI: 1.04 - 1.46), although confidence intervals indicated that the increase in odds may be modest. Other factors considered in the model, including training, clinical experience, and self-perceived knowledge, were not clearly associated with knowledge scores, as shown by the 95% CIs that included 1 (Table 3).

**Table 3:** Association between correct responses to knowledge questions and profession, health facility type, snakebite management training, clinical experience, prior experience treating snakebite cases, and self-perceived knowledge.

		Univariate model	Multivariate model
		Odds Ratio (95% CI)	
Age group	20 - 29	Reference	Reference
	30 - 39	1.19 (0.95, 1.49)	1.25 (0.96, 1.63)
	40 - 49	1.10 (0.88, 1.37)	1.24 (0.88, 1.76)
	≥ 50	0.98 (0.77, 1.25)	1.15 (0.79, 1.67)
Profession	Doctor	Reference	Reference
	Nurse	0.73 (0.60, 0.89)	0.78 (0.62, 0.98)
	Nurse assistant	0.64 (0.53, 0.76)	0.68 (0.55, 0.84)
Health facility	Primary	Reference	Reference
	Secondary	1.17 (1.00, 1.37)	1.23 (1.04, 1.46)
Training	None	Reference	Reference
	During studies only	1.11 (0.95, 1.30)	1.04 (0.87, 1.23)
	Post-graduate	1.00 (0.82, 1.22)	0.89 (0.72, 1.11)
Clinical experience (years)	≤ 1	Reference	Reference
	2 - 5	1.06 (0.78, 1.43)	0.89 (0.64, 1.23)
	6 - 10	1.30 (0.96, 1.76)	1.00 (0.70, 1.43)
	> 10	0.99 (0.75, 1.31)	0.85 (0.58, 1.25)
Treated a snakebite	No	Reference	Reference
	Yes	1.20 (1.04, 1.38)	1.08 (0.90, 1.30)
Self-perceived snakebite management knowledge	No knowledge	Reference	Reference
	Poor	1.20 (0.95, 1.50)	1.10 (0.86, 1.42)
	Medium	1.24 (1.00, 1.73)	1.13 (0.88, 1.44)
	Good	1.34 (1.04, 1.73)	1.15 (0.84, 1.58)
	Excellent	0.72 (0.29, 1.71)	0.70 (0.28, 1.69)

CI: confidence interval.

**Fig 2:**
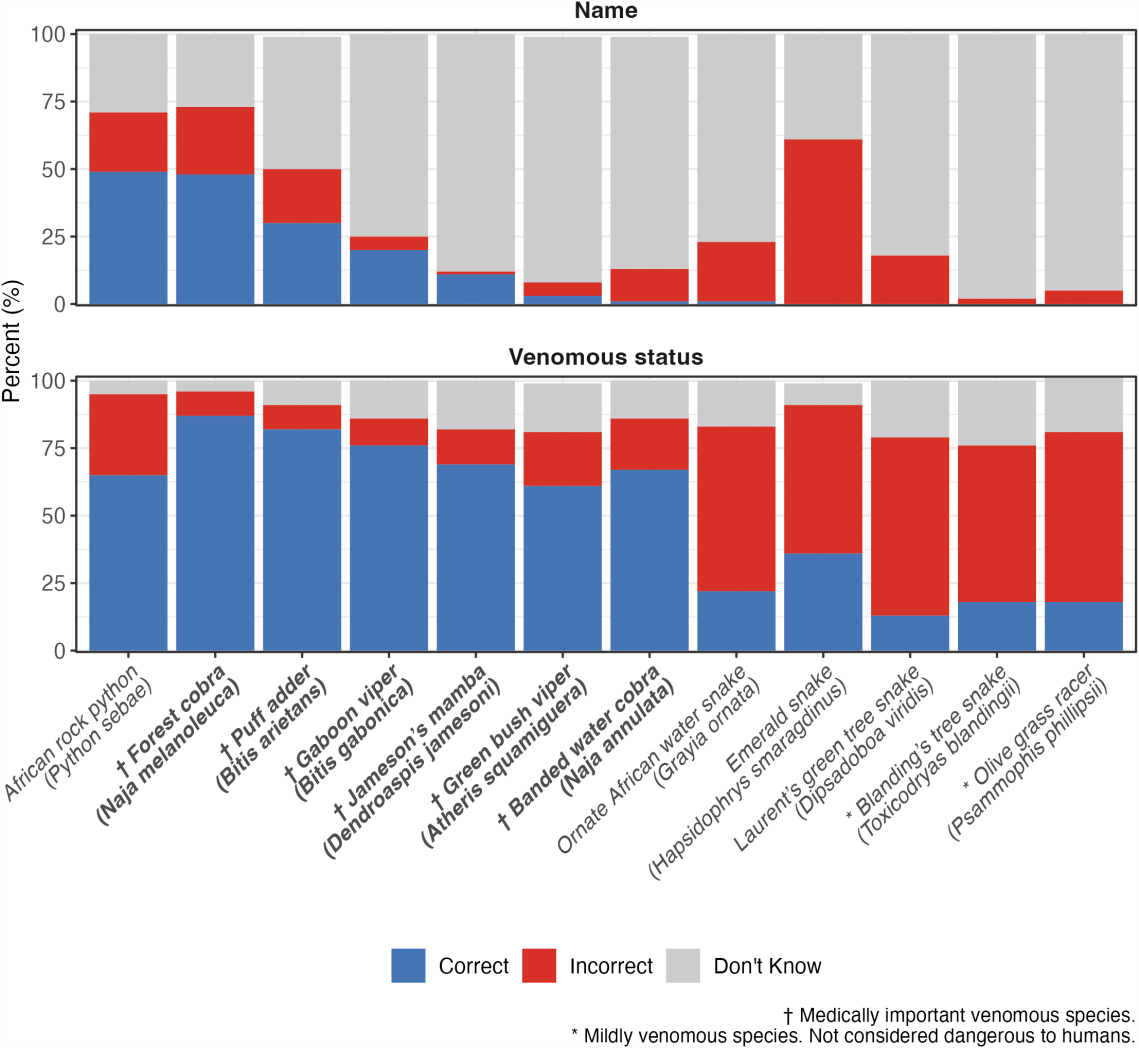
Healthcare workers’ knowledge of snake names and venomous status. Responses to snake identification questions were summarised for each snake as correct (blue), incorrect (red) or don’t know (grey). Venomous snake species are indicated on the x-axis in bold. Snake names provided by healthcare workers were classified as correct if the appropriate local, common, or scientific name was given, as incorrect if a different species name was provided, and as “don’t know” if the response was left blank.

A total of 148 (87%) healthcare workers agreed to see photos of snakes for identification. The snakes most commonly identified correctly by name were the python (*Python sebae*) (49%, 73/148) and forest cobra (*Naja melanoleuca*) (48%, 71/148) (Fig 2).

On average, venomous snakes were correctly identified as venomous in 74% (109/148) of responses. The proportion of incorrect/don’t know answers varied by snake, from 13% (19/148) not identifying the forest cobra as venomous, to 38% (57/148) not identifying the green bush viper as venomous. On average, non-venomous snakes were correctly identified as non-venomous in 28% (42/148) of responses. Incorrect/“don’t know” responses ranged from 35% (52/148) not identifying the African rock python as non-venomous, to 87% (129/148) not identifying the Laurent’s green tree snake as non-venomous. Ten healthcare workers (7%) identified all snakes as venomous.

Overall, the Emerald snake was the most commonly misidentified snake, with 61% (91/148) of names provided being incorrect and the remaining 39% (57/148) being “don’t know”. When a name was provided, the most common incorrect responses were green snake (54%, 49/91) and mamba (38%, 35/91). As well as being the most commonly correctly identified venomous snake (48%, 71/148), the forest cobra was also the most commonly misidentified venomous snake, with 25% (37/148) of names provided being incorrect, and 27% (40/148) being “don’t know”. The most common incorrect responses were black snake (41%, 15/37) and mamba (35%, 13/37).

Overall, 18% (29/165) of healthcare workers reported being aware of published clinical guidelines or treatment protocols. Of these, 59% (17/29) reported that these protocols were used at their workplace, and 59% (10/17) of those indicated that the protocols were available to all staff. When asked to provide details on which published guidelines or treatment protocols they were aware of, 19 (66%) provided details, with 68% (13/19) referring to the package insert for the available antivenom. The remaining protocols included a “hospital protocol”, two textbooks on snake venoms and envenomation and one textbook on medical emergencies, as well as protocols for attaching a tourniquet. No reference was made to local, national, or WHO snakebite management guidelines.

When provided with a set of options for where they would seek guidance when managing snakebite cases, the majority of healthcare workers (67%, 115/171) said they would approach a senior staff member at their facility (Table 4). A total of 34 (20%) health workers indicated that they would seek guidance from a specialised snakebite team, a resource not available in the country at the time of the survey.

**Table 4:** Where healthcare workers would seek guidance in managing snakebites.

	Overall	Primary health facility	Secondary health facility
	% (n/N)		
Colleagues	35.1 (60/171)	29.2 (14/48)	37.4 (46/123)
A senior staff member	67.3 (115/171)	54.2 (26/48)	72.4 (89/123)
Staff at another facility	16.4 (28/171)	33.3 (16/48)	9.8 (12/123)
Specialised snakebite team	19.9 (34/171)	18.8 (9/48)	20.3 (25/123)
Clinical guidelines	16.4 (28/171)	8.3 (4/48)	19.5 (24/123)
Manual/handbook	19.3 (33/171)	12.5 (6/48)	22.0 (27/123)
Internet	30.4 (52/171)	27.1 (13/48)	31.7 (39/123)
Other	7.6 (13/171)	12.5 (6/48)	5.7 (7/123)

Response options were provided to healthcare workers. Multiple could be selected.

Overall, 64% (110/171) of healthcare workers reportedly knew where they could transfer a snakebite patient to. The majority of healthcare workers based at primary health facilities (85%, 39/46) said that they would transfer a snakebite patient to one of the two secondary health facilities within the department. Overall, a similar proportion of healthcare workers from the secondary health facilities reported that they would transfer a snakebite patient either to a hospital outside the department (45%, 26/58), or to one within the department (43%, 25/58), which included either transfer to another unit of the same hospital or transfer to the only other hospital within the department. Details on where health workers stated they would transfer patients to are provided in Table C in S2 File.

## Discussion

Prompt and effective treatment is a key determinant of the clinical outcome for patients bitten by venomous snakes [4,27]. However, this study identified important gaps in knowledge among healthcare workers in Ogooué et des Lacs, Gabon, regarding snakebite symptoms, appropriate management, and the identification of local snake species. These knowledge gaps present a significant barrier to the provision of timely and effective care and must be addressed to improve clinical outcomes.

Knowledge gaps were particularly evident when it came to questions on unrecommended snakebite management practices, with only a small percentage of healthcare workers answering them correctly. This is especially concerning, given that these gaps appear to translate into clinical practice, as our previous retrospective analysis of snakebite cases treated at the same facilities found widespread use of suboptimal treatments [22]. For example, antivenom was administered in a high proportion of cases, including those with only mild signs of envenoming [22]. Unnecessary administration of antivenom increases the risk of allergic reactions, causes high costs for patients, and wastes an often scarce resource. In addition, the lack of knowledge about antivenom dosage likely contributed to individuals receiving doses below the manufacturer’s recommended level [22]. Knowledge assessments further revealed that many health workers were unaware that children should receive the same antivenom dose as adults, which may put this vulnerable group at higher risk of underdosing. Potentially harmful treatment practices, such as the application of tourniquets and the use of venom extractors, were also often identified by health workers as appropriate treatment responses despite evidence that their use increases the risk of severe local complications [28,29]. Differences in overall snakebite knowledge were observed across healthcare worker professions. While doctors had higher odds of answering knowledge questions correctly, it is important to interpret these differences in light of role-specific responsibilities. Nurse assistants, who typically support rather than lead clinical decision-making in snakebite management, would not be expected to have the same knowledge as doctors. Their lower scores, therefore, likely reflect differences in training and scope of practice. Notably, nurses demonstrated greater knowledge in certain areas, such as antivenom dosing, highlighting that expertise can vary according to practical responsibilities. Overall, disparities between professions likely stem from differences in training opportunities and exposure to snakebite cases and should be understood within these professional contexts.

Limited knowledge of appropriate snakebite management practices among healthcare workers has also been reported elsewhere [30–32]. However, evidence suggests that targeted training programs can improve snakebite management [33,34] and increase the number of patients seeking care at formal health facilities [23,35]. Still, important barriers to participation in training exist. As noted in previous studies, training offered during regular working hours can limit participation [36], particularly in understaffed facilities. To maximise accessibility and impact, training must be made accessible to healthcare workers in rural areas and delivered in formats that accommodate clinical responsibilities. Training should also be comprehensive and regularly delivered, covering various aspects, from first aid and recognition of symptoms requiring urgent referral, to antivenom administration and the management of complications. Despite the potential impact of in-depth training programs, our analysis suggests that current training efforts may be insufficient and do not significantly improve knowledge. Only half of the surveyed healthcare workers in Ogooué et des Lacs had received snakebite management training, either during their formal studies or post-graduation. Only one in four doctors had been trained to manage adverse reactions to antivenom, and one in five healthcare workers reported receiving post-graduate training. The median duration of the training was less than half a day, and we found no significant association between training and knowledge. This emphasises the need for systematic, inclusive, and context-specific snakebite management training for healthcare workers in Ogooué et des Lacs. Nationally, training could initially prioritise facility heads and supervising clinicians, who are then supported to train frontline health workers, using a structured curriculum with validated materials, pre- and post-assessments, and supervision to ensure guideline use and DHIS2 reporting. Such training could be integrated into existing NTD programmes and, over time, into pre-service curricula. In parallel, community health workers should receive basic training on prevention and early referral, and public awareness campaigns could be delivered in high-risk areas. Studies designed to assess the direct impact of training on knowledge scores could help determine whether improved snakebite-specific training effectively addresses the knowledge gaps identified in this study.

Knowledge of local snake species was also generally poor. The python and forest cobra were the most commonly identified species, likely due to their distinctive appearances. While less than 2% correctly identified the other cobra species, the banded water cobra, likely because it was shown in a resting position, without its hood displayed. Healthcare workers sometimes failed to identify venomous snakes and often misidentified non-venomous snakes as venomous. Similar gaps in knowledge of local snakes among healthcare workers have been reported elsewhere [37,38] and may hinder clinical decision-making when patients can describe or show a photo of the snake that bit them [39]. Providing health facilities with visual reference materials, such as posters, and training healthcare workers to recognise the signs and symptoms that indicate the need for antivenom could help reduce unnecessary antivenom use.

When asked where they would seek advice in managing a snakebite case, most healthcare workers said that they would consult a senior colleague. Yet, our findings show that more years of clinical experience and prior experience treating snakebite cases were not associated with a higher odds of answering knowledge questions correctly.

Notably, around 20% of respondents indicated they seek guidance from a “specialised snakebite team”, a resource that does not currently exist in Gabon. It seems some health workers misunderstood this question, responding with where they would seek guidance, rather than where they have already sought it. Still, establishing an expert committee of clinicians who offer real-time advice on snakebite management and treatment, comparable to poison control centres or telemedicine services developed for other neglected tropical diseases [40,41], could significantly improve the management of patients with snakebite envenoming in Gabon and potentially reduce the need for patient transfers to other facilities, which delays treatment. These services have already been successfully rolled out in Malaysia, with the Remote Envenomation Consultation Services (RECS) assisting healthcare providers in the management of envenomations [42].

The awareness of clinical guidelines was limited, with most healthcare workers unfamiliar with any published guidelines for snakebite management, and most health workers only mentioning the manufacturer’s guidelines for antivenom administration. In the absence of national guidelines in Gabon, the WHO guidelines for the African region [43] offer a valuable framework. However, the WHO guidelines have not been updated since 2010 and are not adapted to Gabon’s specific snake fauna or health system constraints. Developing national snakebite management guidelines and updating the WHO regional guidelines in line with the WHO 2019 strategy [1] would provide healthcare workers with a much-needed resource. In areas, such as Ogooué et des Lacs, where relatively few cases are seen within the health system and clinical experience with snakebites may be limited, access to practical, user-friendly guidelines is likely to have the greatest long-term impact on the quality of care [35].

In addition to improved availability of relevant snakebite management guidelines and support, there is an urgent need to improve access to essential resources, such as species-appropriate and affordable antivenom, ventilators, and reliable transportation to health facilities, to better equip healthcare workers in responding effectively to snakebite cases [22,44]. In addition, improving access to essential resources will likely reduce the need for patient transfers between the facilities in the department and to facilities outside the department, thereby reducing potential delays in treatment.

This study is the first to evaluate knowledge of snakebite symptoms, management, and local snake species among healthcare workers in a department of Gabon, highlighting key areas for improvement. Including open-ended questions, rather than relying solely on true/false questions, may have provided a more comprehensive understanding of healthcare workers’ knowledge. In addition, internal consistency measures could have been considered in the design of the questionnaire to strengthen knowledge assessments. A formal training needs assessment could also inform the development of more targeted and effective training programs [36].

While it is possible that some respondents gave misleading responses to some questions due to social desirability bias, especially in small health facilities where health workers may be identifiable from completed questionnaires, we attempted to minimise this risk by asking individuals to complete the questionnaire themselves instead of conducting interviews. In addition, this potential bias does not influence the assessment of health worker knowledge, but rather the assessment of experience and training. However, because health workers in this study reported limited snakebite training and experience, the impact of this potential bias is likely limited.

Although the study was designed to survey all eligible healthcare workers in the department, not all eligible staff were available during data collection, despite repeated visits to facilities, resulting in participation from approximately 78% of the target population. While this means that complete coverage was not achieved, we have no reason to believe that non-participating staff differ systematically from those who participated. This should nonetheless be considered when interpreting the findings.

Although we did not observe a clear association between prior snakebite training and knowledge scores, this study was not designed to formally evaluate the effectiveness of training interventions. Training exposure was measured broadly and did not capture important characteristics such as content, quality, duration, or recency. As a result, the confidence intervals around these estimates indicate uncertainty, and the absence of a statistically significant association should be interpreted cautiously. These findings are therefore best viewed as exploratory within the study setting and should not be interpreted as definitive evidence regarding training effectiveness. Future studies specifically designed to evaluate training effectiveness, with clearly defined training exposures and outcomes, would be valuable to determine which approaches most effectively improve snakebite knowledge and clinical practice in this setting.

All health facilities included in this study are part of the national public health system. Although the findings should be interpreted primarily as representative of the study setting, we have no reason to believe that there are systematic differences between the health workers included in this study and those in other rural departments of Gabon. Nevertheless, studies conducted in other regions of Gabon would be valuable to formally assess the generalisability of these findings, identify any additional knowledge gaps, and inform the design of a comprehensive, nationwide initiative to strengthen snakebite knowledge and management.

## Conclusion

To improve the clinical outcomes for snakebite patients, it is important that health facilities are equipped with sufficient resources and health workers skilled in snakebite management. In this analysis, we found that health workers in the Ogooué et des Lacs department of Gabon lack knowledge of snakebite symptoms, appropriate treatment practices, and the common snakes in the area. To ensure that snakebite patients receive prompt and effective treatment, it is essential that healthcare workers are trained in snakebite management and that the use of the WHO snakebite management guidelines is promoted until local guidelines are established.

## Supporting information

S1 Data STROBE Checklist for Cross-Sectional Studies. von Elm E, Altman DG, Egger M, Pocock SJ, Gøtzsche PC, Vandenbroucke JP. Strengthening the Reporting of Observational Studies in Epidemiology (STROBE) statement: guidelines for reporting observational studies. BMJ. 2007;335(7624):806–8.
(PDF)

S1 File Questionnaire. (PDF)

S2 File Detailed Questionnaire Responses. (PDF)

S3 File Survey Data. (XLSX)
