# Supplementary material for: Snakebite knowledge among healthcare workers in Gabon: A health facility-based cross-sectional survey
Source: PLoS Negl Trop Dis. 2026 Mar 16;20(3):e0013742. doi: 10.1371/journal.pntd.0013742 (PMC12991226; doi:10.1371/journal.pntd.0013742)
Supplement: S1 File — (PDF) [file pntd.0013742.s002.pdf]

S1 File: Questionnaire

Questionnaires for health workers. ID No: ____

A General information

1. Type of questionnaire
   o Printed
   o Digital

2. Date of interview
   o (yyyy/mm/dd)

3. Informed consent given?
   o Yes
   o No

4. Age in years
   ____

5. Gender
   o Male
   o Female

Characteristics of surveyed healthcare workers

6. Professional qualification
   o Head of medical doctors' department
   o Medical doctor
   o Last-year medical student
   o Head of nurses department
   o Assistant nurse
   o Nurse
   o Pharmacist
   o Trainee-nurse. Specify____
   o Midwife
   o Nurse-Midwife
   o Other. Specify____

7. Place of work (Name of the institution)
   o Hôpital Albert Schweitzer, Lambaréné
   o Centre de Santé Urbain, Lambaréné
   o Hôpital Régional Georges Rawiri, Lambaréné
   o Centre hospitalière de Bifoun, Bifoun
   o Dispensaire. Specify (name and localisation) ____
   o Centre de Traitement Ambulatoire, Lambaréné
   o Centre de Recherches Médicales de Lambaréné
   o Other. Specify____

8. Department
   o Emergency
   o Internal medicine
   o Surgery
   o Pediatrics
   o Obstetrics
   o Consultation (polyclinic)
   o Pharmacy
   o No specialisation
   o Other. Specify______

9. Work experience in years (in the medical sector)
   ____

10. Have you been trained in the management of snakebites during your studies?
   o Yes
   o No

10.1 If Yes: How many hours (estimate)
   ____

10.2 What was this training about?
   o Snakes, snake venoms and syndromes of envenoming
   o Prevention of snakebite
   o Diagnosis of snakebite
   o Treatment of snakebite
   o Managing adverse antivenin reactions
   o Other

11. Have you received any additional postgraduate training/ after your studies in the management of snakebites?
   o Yes.
   o No

11.1 If Yes: How many hours (estimate)
   ____

11.2 What was this training about?
   o Snakes, snake venoms and syndromes of envenoming
   o Prevention of snakebite
   o Diagnosis of snakebite
   o Treatment of snakebite
   o Managing adverse antivenin reactions
   o Other. Specify______

12. Are you aware of any (published) guidelines or treatment protocols for managing snakebites?

o Yes. Specify______
o No

13. Do you use guidelines or treatment protocols to manage snakebites in your health facility?
o Yes. Specify______
o No

13.1 Are they available to all staff?
o Yes. Where? ______
o No

14. Have you already treated snakebite patients?
o Yes
o No

14.1 If yes, when?
o < 1 year
o 1-5 years
o >5 years

14.2 If yes, how many patients did you treat?
____

15. How would you describe your knowledge of snakebite management?
o Excellent
o Good
o Medium
o Poor
o No knowledge

16. Where, if at all, do you get guidance or support when managing a patient with snakebite? Please select all that apply.
o Ask colleagues in your place of work
o Ask the superior in your place of work
o Consult staff at another facility
o Consult specialist snakebite team
o Clinical/ official guidelines on snakebite management
o In a handbook
o On the internet
o Other. Specify_____

17. Do you know to which centre you could transfer the patient?
o Yes. Specify (Name and location) _____
o No

B General and snake-specific knowledge

Symptoms after snakebite can be (amongst others)

1. Neurotoxic (involvement of the central nervous system: muscle paralysis and brain damage)
   o True
   o False

2. Hematotoxic (coagulation disorders and systemic bleeding)
   o True
   o False

3. Cytotoxic (tissue damage and edema)
   o True
   o False

4. Myelotoxic (bone marrow depression and decreased blood cell production)
   o True
   o False

5. The Ptosis (paralysis of the eyelid) is a common symptom in neurotoxic snakebite envenomings.
   o True
   o False

6. The Swelling of the bitten limb is a common symptom after snakebite.
   o True
   o False

7. The Bleeding of gums is a possible symptom in snakebite envenomings.
   o True
   o False

8. The so-called “dry bites”, bites by venomous snakes that do not lead to any symptoms except fangmarks, are frequent - A venomous snake can bite without injecting venom.
   o True
   o False

9. After a bite from a forest cobra bite, haemorrhage/ systemic bleeding is a common symptom.
   o True
   o False

10. After a bite from a gaboon viper, haemorrhage/ systemic bleeding is a common symptom.

o True
o False

11. After a bite from a python, neurotoxic signs, including respiratory disorders, are frequent.
    o True
    o False

12. After a bite from a blandings tree snake, cytotoxic lesions and necrosis are common symptoms.
    o True
    o False

C Management of snakebites

1. If the patient shows signs of local necrosis/ gangrene after a snakebite, surgical debridement of the dead tissue is contraindicated.
   o True
   o False

2. The Prothrombin time or the 20 minutes whole blood clotting test (tube sec) are recommended to assess severity of hematotoxic envenomation.
   o True
   o False

3. A liver function test has to be performed immediately after the snakebite to assess necessity of antivenom.
   o True
   o False

4. A tourniquet is a recommended first aid measurement in case of a bitten limb.
   o True
   o False

5. The immobilization of the patient and the bitten limb and transport to a health center is recommended in all snakebite patients.
   o True
   o False

6. The suction of the venom by specific vacuum pumps, if available, is indicated after a snakebite.
   o True
   o False

7. The non-steroidal anti-inflammatory drugs such as ibuprofen or diclofenac are an excellent pain killer and equivalent to paracetamol in case of a snakebite.
   o True
   o False

8. There is an absolute contraindication to administer drugs such as morphine, fentanyl, tramadol or other opioids to snakebite victims.
   o True
   o False

9. All patients who have been bitten by a venomous snake need an antivenom.
   o True
   o False

10. The anaphylactic/allergic shock is a serious and frequent side effect of antivenom.
    o True
    o False

11. The adrenalin is the most important drug for treating an anaphylactic/allergic shock after the administration of an antivenom.
    o True
    o False

12. The children are given the same dose of antivenom as adults.
    o True
    o False

<u>D Identification of snakes (based on photos shown to respondents)</u>
In the following part, pictures of snakes will be shown. Do you agree?
o Yes
o No

For each snake:
o Venomous
o Not/ little venomous

Have you ever seen the snake?
o Yes
o No

Do you know the name of the snake?
o Yes
o No

If yes, Specify_____________________

Comments on the snake
____

Answer key to snake identification questions (not included in questionnaire)

Table A. Details of snake species shown in photos for identification.

Snake photo #	Name	Venomous status
1	Puff Adder (*Bitis arietans*)	Yes
2	Banded water cobra (*Naja annulata*)	Yes
3	Forest cobra (*Naja melanoleuca*)	Yes
4	Gaboon viper (*Bitis gabonica*)	Yes
5	Emerald snake (*Hapsidophrys smaragdinus*)	No
6	Ornate African water snake (*Grayia Ornata*)	No
7	Green bush viper (*Atheris squamiguera*)	Yes
8	Olive grass racer (*Psammophis phillipsii*)	No*
9	Blanding’s tree snake (*Toxicodryas blandingii*)	No*
10	African rock python (*Python sebea*)	No
11	Jameson’s mamba (*Dendroaspis jamesoni*)	Yes
12	Laurent’s green tree snake (*Dipsadoboa viridis*)	No

* Mildly venomous. Not considered dangerous to humans.
