# Supplementary material for: Snakebite knowledge among healthcare workers in Gabon: A health facility-based cross-sectional survey
Source: PLoS Negl Trop Dis. 2026 Mar 16;20(3):e0013742. doi: 10.1371/journal.pntd.0013742 (PMC12991226; doi:10.1371/journal.pntd.0013742)
Supplement: S2 File — (PDF) [file pntd.0013742.s003.pdf]

S2 File: Detailed Questionnaire Responses

Table A. Training topics covered according to when training was received and profession.

		Overall	Doctor	Nurse	Assistant Nurse
		% (n/N)[1]			
During studies	Snakes, snake venom, and envenoming syndromes	53.3 (40/75)	68.4 (13/19)	48.1 (13/27)	48.3 (14/29)
	Prevention	45.3 (34/75)	42.1 (8/19)	48.1 (13/27)	44.8 (13/29)
	Diagnosis	54.7 (41/75)	68.4 (13/19)	55.6 (15/27)	44.8 (13/29)
	Treatment	80.0 (60/75)	68.4 (13/19)	85.2 (23/27)	82.8 (24/29)
	Adverse antivenom reactions	28.0 (21/75)	36.8 (7/19)	22.2 (6/27)	27.6 (8/29)
Post-graduate	Snakes, snake venom, and envenoming syndromes	39.4 (13/33)	62.5 (5/8)	20.0 (1/5)	35.0 (7/20)
	Prevention	27.3 (9/33)	25.0 (2/8)	20.0 (1/5)	30.0 (6/20)
	Diagnosis	45.5 (15/33)	50.0 (4/8)	40.0 (2/5)	45.0 (9/20)
	Treatment	72.7 (24/33)	87.5 (7/8)	60.0 (3/5)	70.0 (14/20)
	Adverse antivenom reactions	30.3 (10/33)	37.5 (3/8)	40.0 (2/5)	25.0 (5/20)

[1] Denominator used was the total number of healthcare workers by profession who received snakebite training during their studies or post-graduation.

Table B. Details on correct responses to knowledge questions.

		Correct Response	Overall	Doctor	Nurse	Nurse assistant
		% (n/N)				
**Assessment: snakebite symptoms**						
Symptoms after a snakebite can include	Neurotoxic	True	85.4 (146/171)	90.3 (28/31)	84.3 (43/51)	84.3 (75/89)
	Hemotoxic	True	66.7 (114/171)	80.6 (25/31)	76.5 (39/51)	56.2 (50/89)
	Cytotoxic	True	67.8 (116/171)	87.1 (27/31)	68.6 (35/51)	60.7 (54/89)
	Myelotoxic	False	72.5 (124/171)	61.3 (19/31)	76.5 (39/51)	74.2 (66/89)
Common symptoms after snakebites include	Ptosis	True	53.2 (91/171)	45.2 (14/31)	47.1 (24/51)	59.6 (53/89)
	Swelling of bitten limb	True	90.6 (155/171)	100 (31/31)	94.1 (48/51)	85.4 (76/89)

Bleeding gums is a possible symptom after a snakebite	True	30.4 (52/171)	64.5 (20/31)	25.5 (13/51)	21.3 (19/89)
Dry bites from venomous snakes are common	True*	46.2 (79/171)	61.3 (19/31)	47.1 (24/51)	40.4 (36/89)
Systemic bleeding after a forest cobra bite is common	False	37.4 (64/171)	29.0 (9/31)	43.1 (22/51)	37.1 (33/89)
Systemic bleeding after a Gaboon viper bite is common	False*	35.1 (60/171)	29.0 (9/31)	33.3 (17/51)	38.2 (34/89)
Neurotoxic signs including respiratory disorders after a python bite are common	False	44.4 (76/171)	48.4 (15/31)	49.0 (25/51)	40.4 (36/89)
Cytotoxic lesions and necrosis after a Blanding's tree snake bite are common	False	30.4 (52/171)	22.6 (7/31)	33.3 (17/51)	31.5 (28/89)
**Assessment: snakebite management**					
Debridement contraindicated in cases with necrosis/gangrene	False	60.8 (104/171)	67.7 (21/31)	51.0 (26/51)	64.0 (57/89)
Prothombin time or the 20-minute WBCT are recommended to assess hemotoxic envenoming	True	84.2 (144/171)	100 (31/31)	88.2 (45/51)	76.4 (68/89)
A liver function test has to be performed immediately after a snakebite to assess the need for antivenom	False	24.0 (41/171)	35.5 (11/31)	23.5 (12/51)	20.2 (18/89)
A tourniquet is a recommended first-aid measure on the bitten limb	False	29.8 (51/171)	58.1 (18/31)	33.3 (17/51)	18.0 (16/89)
Immobilization and transport to healthcare centre is recommended for all snakebite patients	True	95.3 (163/171)	93.5 (29/31)	96.1 (49/51)	95.5 (85/89)
Venom suction using vacuum pumps is indicated after a snakebite	False	26.9 (46/171)	35.5 (11/31)	19.6 (10/51)	28.1 (25/89)
Non-steroidal anti-inflammatory drugs (NSAIDs) are good pain killers and are equivalent to paracetamol for snakebites	False	29.8 (51/171)	32.3 (10/31)	33.3 (17/51)	27.0 (24/89)
There is absolute contraindication to give opioids to snakebite victims	False	38.6 (66/171)	58.1 (18/31)	33.3 (17/51)	34.8 (31/89)
All patients bitten by venomous snakes need antivenom	False	15.2 (26/171)	41.9 (13/31)	9.8 (5/51)	9.0 (8/89)
Anaphylactic/allergic shock is a common and serious side effect of antivenom	True	74.9 (128/171)	77.4 (24/31)	78.4 (40/51)	71.9 (64/89)

Adrenaline is the most important medication for treating anaphylactic/allergic shock after antivenom administration	True	63.7 (109/171)	77.4 (24/31)	74.5 (38/51)	52.8 (47/89)
Children receive the same dose of antivenom as adults	True	39.8 (68/171)	29.0 (9/31)	41.2 (21/51)	42.7 (38/89)

* Correct response was determined through consultation with local experts. However, due to a lack of clear scientific evidence, this question was not included in the calculation of symptom or overall knowledge scores.

Table C. Where healthcare workers would transfer a snakebite patient to, by health facility type and profession.

	Primary health facility			Secondary health facility		
	Doctor	Nurse	Assistant Nurse	Doctor	Nurse	Assistant Nurse
	% (n/N)					
Hospital in the department	57.1 (4/7)	93.3 (14/15)	87.5 (21/24)	23.1 (3/13)	55.6 (10/18)	44.4 (12/27)
Hospital outside the department	28.6 (2/7)	6.7 (1/15)	4.2 (1/24)	61.5 (8/13)	27.8 (5/18)	48.1 (13/27)
Medical centre	14.3 (1/7)	0.0 (0/15)	0.0 (1/24)	0.0 (0/13)	5.6 (1/18)	0.0 (0/27)
Unclear	0.0 (0/7)	0.0 (0/15)	4.2 (1/24)	7.7 (1/13)	11.1 (2/18)	7.4 (2/27)
Other[1]	0.0 (0/7)	0.0 (0/15)	4.2 (1/24)	7.7 (1/13)	0.0 (0/18)	0.0 (0/27)

[1] Other includes "CERMEL" and "military hospital"

Primary health facilities include 22 dispensaries, four medical centres, and one outpatient clinic.

Secondary health facilities include two hospitals.
